# Dynamic Analysis of Vascular Morphogenesis Using Transgenic Quail Embryos

**DOI:** 10.1371/journal.pone.0012674

**Published:** 2010-09-14

**Authors:** Yuki Sato, Greg Poynter, David Huss, Michael B. Filla, Andras Czirok, Brenda J. Rongish, Charles D. Little, Scott E. Fraser, Rusty Lansford

**Affiliations:** 1 Division of Biology, Biological Imaging Center, Beckman Institute, California Institute of Technology, Pasadena, California, United States of America; 2 Department of Anatomy and Cell Biology, University of Kansas Medical Center, Kansas City, Kansas, United States of America; Katholieke Universiteit Leuven, Belgium

## Abstract

**Background:**

One of the least understood and most central questions confronting biologists is how initially simple clusters or sheet-like cell collectives can assemble into highly complex three-dimensional functional tissues and organs. Due to the limits of oxygen diffusion, blood vessels are an essential and ubiquitous presence in all amniote tissues and organs. Vasculogenesis, the de novo self-assembly of endothelial cell (EC) precursors into endothelial tubes, is the first step in blood vessel formation [1]. Static imaging and in vitro models are wholly inadequate to capture many aspects of vascular pattern formation in vivo, because vasculogenesis involves dynamic changes of the endothelial cells and of the forming blood vessels, in an embryo that is changing size and shape.

**Methodology/Principal Findings:**

We have generated Tie1 transgenic quail lines Tg(*tie1*:H2B-eYFP) that express H2B-eYFP in all of their endothelial cells which permit investigations into early embryonic vascular morphogenesis with unprecedented clarity and insight. By combining the power of molecular genetics with the elegance of dynamic imaging, we follow the precise patterning of endothelial cells in space and time. We show that during vasculogenesis within the vascular plexus, ECs move independently to form the rudiments of blood vessels, all while collectively moving with gastrulating tissues that flow toward the embryo midline. The aortae are a composite of somatic derived ECs forming its dorsal regions and the splanchnic derived ECs forming its ventral region. The ECs in the dorsal regions of the forming aortae exhibit variable mediolateral motions as they move rostrally; those in more ventral regions show significant lateral-to-medial movement as they course rostrally.

**Conclusions/Significance:**

The present results offer a powerful approach to the major challenge of studying the relative role(s) of the mechanical, molecular, and cellular mechanisms of vascular development. In past studies, the advantages of the molecular genetic tools available in mouse were counterbalanced by the limited experimental accessibility needed for imaging and perturbation studies. Avian embryos provide the needed accessibility, but few genetic resources. The creation of transgenic quail with labeled endothelia builds upon the important roles that avian embryos have played in previous studies of vascular development.

## Introduction

One of the least understood and most central questions confronting biologists is how initially simple clusters or sheet-like cell collectives can assemble into highly complex three-dimensional functional tissues and organs. Due to the limits of oxygen diffusion, blood vessels are an essential and ubiquitous presence in all amniote tissues and organs. Vasculogenesis, the de novo self-assembly of endothelial cell (EC) precursors into endothelial tubes, is the first step in blood vessel formation. The primary vascular plexus develops from mesodermal cells that differentiate into primordial endothelial cells and assemble into an interconnected tubular network [2], [3], [4], [5]. The vascular precursor cells are initially scattered throughout the mesoderm; they subsequently assemble locally or move to the site of a developing vessel [6], [7], [8] [5] and then differentiate into hematopoietic cells or ECs [9], [10]. The endothelia of the dorsal aortae, the yolk sac vessels, and other major vessels all undergo vasculogenesis [2], [8] prior to the onset of circulation. Primordial endothelial/endocardial tubes in the bilaterally located anterior lateral plate mesoderm [11] fuse at the midline to form the lining of the linear heart tube at stage 8/9 [12]. Data from several different lines of research have suggested that vasculogenesis is modulated not only by cell-cell and cell–extracellular matrix (ECM) interactions but also by growth factors and morphogens [1], [2], [13], [14], [15]. ECs are the primary integrator of the mechanical and chemical cues that guide vascular wall physiology and pathology [16], requiring that any analysis of vascular development have single-cell resolution.

Vascular patterning is complex; understanding this complexity requires time-resolved analyses across wide time and length scales. Static imaging and *in vitro* models are wholly inadequate to capture many aspects of vascular pattern formation *in vivo*, because vasculogenesis involves dynamic changes of the endothelial cells and of the forming blood vessels, in an embryo that is changing size and shape. The avian embryo develops a human-like four-chambered heart as true for all warm-blooded vertebrates. Unlike mice, the accessibility of the avian embryos makes them excellent specimens for experimental embryology [9], [17] and time-lapse microscopy [18], [19]. However, until recently, the genetic techniques that have been so powerfully exploited in mice, have not been available in avians.

The discrepancy between the best amniote animal models for molecular studies (mouse) versus live imaging (avians), motivated us to construct transgenic, fluorescent protein (FP) expressing Coturnix quail as an experimental system using lentiviral vectors [20], [21], [22], [23]. Lentiviral vectors permit efficient and long lasting gene transfer into a variety of cell types [24], [25], [26], as well as the germ cell lineage [25], [27]. Incorporating normal cis-regulatory domains in the vector confers tissue specific gene expression. Coturnix quail offer advantages in the small size of its egg, the moderate size of the breeding adults, and its short generation time.

To increase our understanding of how endothelial cells and their precursors are precisely patterned in space and time, we generated Tg(*tie1*:H2B-eYFP) (enhanced YellowFP) quail embryos that express H2B-eYFP in all endothelial tubes and the endocardium. Our analysis reveals striking cell- and tissue-level events, including cell division, cell differentiation and tissue displacements that provide novel insights into how warm-blooded vertebrates form a primary vascular network.

## Results

### Tie1:H2B-YFP is expressed specifically in endothelial cells

**Figure 1:**
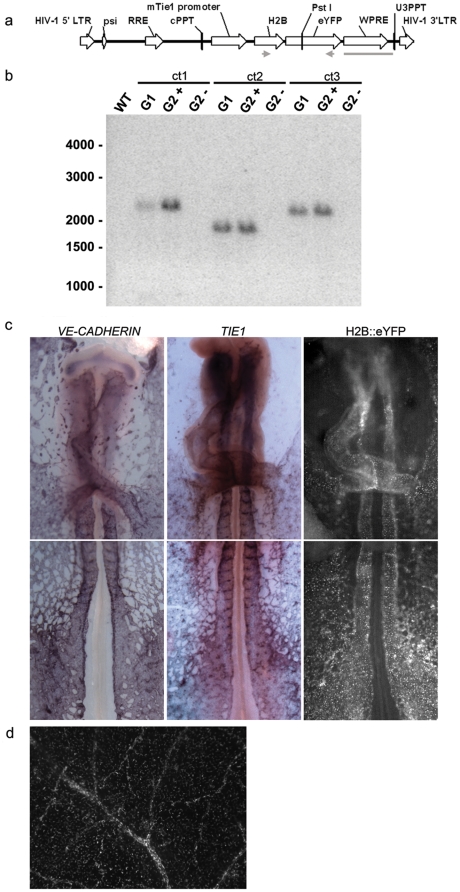
Molecular characterization of Tg(*tie1*:H2B-eYFP) quail. (a) Schematic representation of the *tie1*:H2B-eYFP lentivector following chromosomal integration. The length of the proviral sequence from the 5′ LTR to the 3′ LTR is 4340 bp. The gray arrows represent the location of the PCR primers used to screen G1 hatchlings for the transgene. These primers amplified an 859 bp sequence that spanned the junction of H2B and eYFP DNA regions. The *Pst*I restriction site used to digest the genomic DNA for Southern blotting analysis is indicated. The gray line represents the 646 bp probe used during Southern analysis. LTR, long terminal repeat; psi, packaging signal; RRE, Rev-response element; cPPT, central polypurine tract; WPRE, woodchuck hepatitis virus posttranscriptional regulatory element. (b) Screening hatchling genomic DNA by Southern blot analysis. Genomic DNA isolated from the chorioallantoic membrane of the eggshell of G1 quail was digested with *Pst*I, which cut once inside the transgene, separated by gel electrophoresis and transferred to a nylon membrane. The blot was hybridized with a 646 bp ^32^P-labeled probe designed to the WPRE element within the transgene. The blot shows three positive transgenic G1 hatchlings (Tg(*tie1*:H2B-eYFP^ct^; ct1, ct2, ct3) from the single founder (G0) breeding pair. All three show single integrations at distinct sites within the genome (ct1, 2400 bp; ct2, 1900 bp; ct3 2200 bp). When bred to a WT, these G1 birds produced phenotypically positive G2 hatchlings (G2^+^) about 50% of the time as expected. Genomic DNA from phenotypically negative hatchlings lacked the transgene (G2^−^). (c) In situ hybridizations confirm *tie1* driven H2B-EYFP expression in ECs. *VE-CADHERIN* mRNA expression. *TIE1* mRNA expression. H2B-eYFP protein expression. (d) Fluorescence dissecting microscope acquired image of H2B-eYFP+ ECs seen within the blood vessels of CAM within shell from the offspring of a Tg(*tie1*:H2B-eYFP) founder quail.

HIV based VSV-g pseudotyped lentiviruses that encode H2B-eYFP under the transcriptional control of the mouse *TIE1* promoter (Fig. 1a) were injected into the subgerminal space of stage X [28] Coturnix blastoderm cells. The *TIE1* gene encodes a receptor tyrosine kinase that is specifically expressed in the vascular endothelial lineage [29], [30]. The injected embryos were incubated to hatching, grown and bred with wild-type quail [23]. Putative transgenic founders were initially screened using DNA isolated from feather blood cells by PCR and Southern blot analysis for the eYFP gene (Fig. 1b). Southern blot analysis revealed that each line contains a single copy of the transgene at unique integration sites (Fig. 1b). Conveniently, the Tg(*tie1*:H2B-eYFP)^+^ offspring express the transgene strongly in the chorioallantoic membrane (CAM) blood vessels of freshly hatched eggs and are thereby easy to identify using an epifluorescence stereomicroscope (Fig. 1d). We established stable breeding colonies for three independent Tg(*tie1*:H2B-eYFP) quail lines (identified as *q1, q2, and q3*).

**Figure 2:**
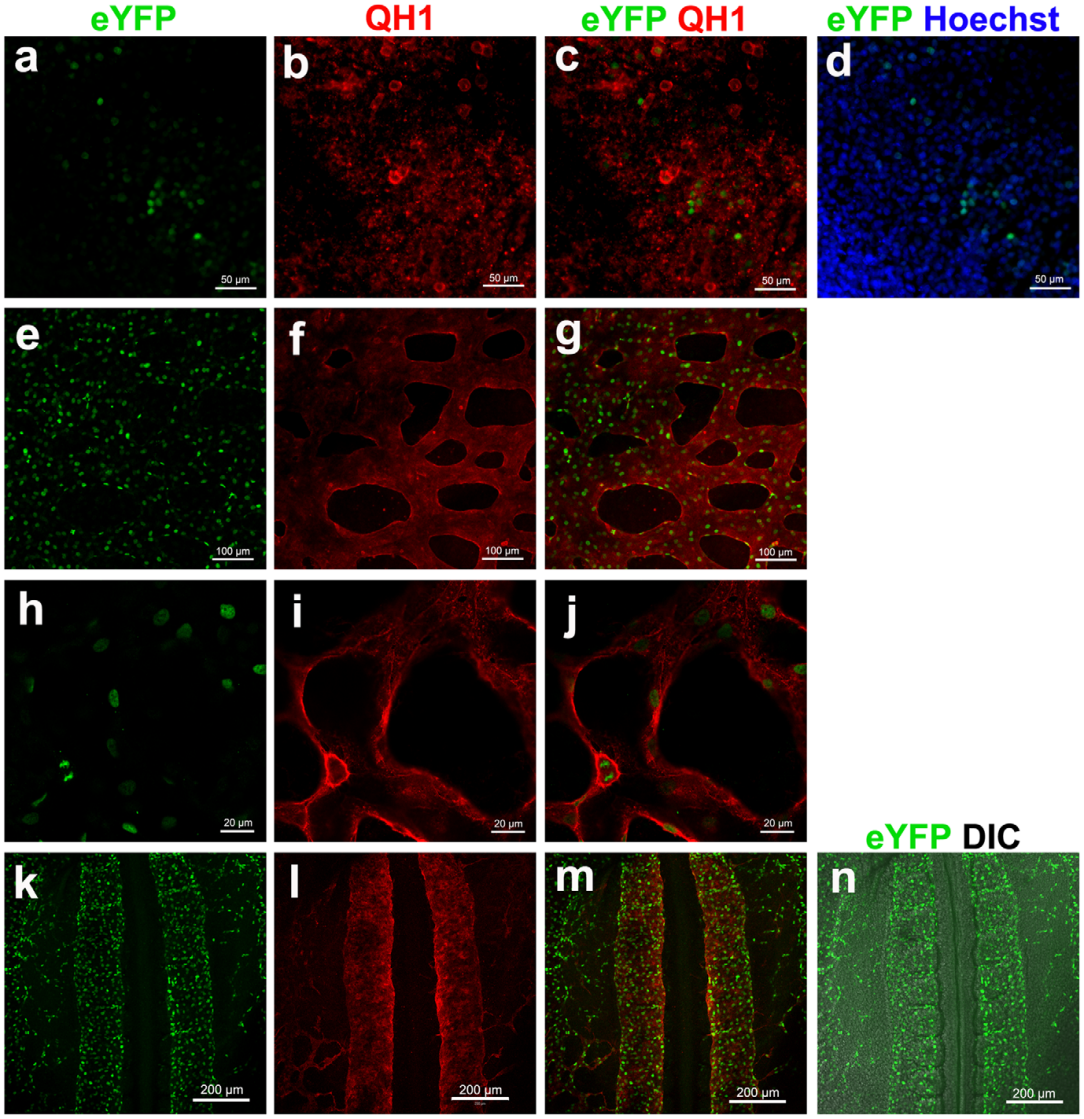
H2B-eYFP signals in the Tg(*tie1*:H2B-eYFP) quail embryos highlight individual ECs. (a–d) Extraembryonic area of a Tg(*tie1*:H2B-eYFP) embryo at HH stage 6 (HH6). H2B-eYFP signals (a) co-localizing with Hoechst signals (d) are found in QH1^+^ area (red in b and d). (e–g) Extraembryonic vascular plexus in a Tg(*tie1*:H2B-eYFP) embryo at HH11. (h–j) A high magnification confocal slice image of the vascular plexus exhibits the H2B-eYFP signals (h) are localized inside of the QH1-signals demarcating every endothelial cell membrane (red in i and j). (k–n) Dorsal aortae at HH stage 11. Anterior is top. Posterior is bottom. (a–g and k–n) Z-stacked images. (h–j) Confocal section images.

H2B-eYFP expression is first observed at late HH stage 6 in the ECs within extraembryonic blood islands, the earliest discernable vascular structures in avian embryos [31], [32], concomitant with the onset of *TIE1* mRNA expression (Fig. 1c and 2a). The expression patterns of Tg(*tie1*:H2B-eYFP) embryos for HH stages 6–12 (Somite stage (ss) 0–12) match the endogenous expression patterns of EC restricted *TIE1* and *VE-CADHERIN* mRNA, including all endothelial tubes and the endocardium (Fig. 1c). Furthermore, the H2B-eYFP^+^ cells in all three transgenic lines are immunoreactive with the QH1 antibody, a reference standard for the vascular endothelial lineage in quail [33](Fig. 2). These data confirm that the Tg(*tie1*:H2B-eYFP) transgenic animals recapitulate the endogenous *TIE1* expression pattern and are entirely consistent with an EC lineage fate.

All three Tg(*tie1*:H2B-eYFP) quail lines fluorescently mark ECs in a specific and heritable manner, yet show subtle variations among the lines that likely reflects differences in the chromosomal integration site of the transgene [34]. H2B-eYFP expression first appears at late HH stage 6 in the extra-embryonic blood islands in Tg(*tie1*:H2B-eYFP)^q1^ and Tg(*tie1*:H2B-eYFP)^q2^; whereas H2B-eYFP^+^ cells are not observed until stage 9 for Tg(*tie1*:H2B-eYFP)^q3^. During early development, Tg(*tie1*:H2B-eYFP)^q2^ expresses H2B-eYFP at the highest relative fluorescence; Tg(*tie1*:H2B-eYFP)^q1^ and Tg(*tie1*:H2B-eYFP)^q3^ display approximately 30% and 50% less. The fluorescence expression patterns are consistent within each transgenic line throughout successive generations and between specimens. All three lines are sufficiently bright for dynamic imaging studies, and yielded indistinguishable results in both the embryonic and extraembryonic tissues.

### Time-lapse imaging of Tg(*tie1*:H2B-YFP) quail embryos

Vasculogenesis occurs over the course of hours to days in and around the developing embryo. Accordingly, we imaged Tg(*tie1*:H2B-eYFP) quail embryos over time (i.e. 12–26 hours) through their ventral surfaces in a modified New culture system [44], which permits dynamic imaging of whole embryos at single cell resolution[19]. To follow the large-scale cell and tissue movements within the developing embryo (Videos S1–S4), we acquired over 100 differential interference contrast (DIC) and fluorescence movies with 5×, 10×, and 20× objectives. In previous studies [5], [8], vasculogenesis and tissue motions were imaged by *in vivo* labeling of ECs with QH1 antibodies tagged with fluorescent dyes. *In vivo* imaging of the Tg(*tie1*:H2B-YFP) permits such analyses to be carried to the cellular level more reliably; the H2B-eYFP^+^ ECs could be identified clearly and tracked longer within the endothelial tubes (Videos S2, S3). The H2B-eYFP nuclear localization marker is ideal for imaging cellular displacements and cell division [36]. Tracking a bright well-defined object (nucleus) permits resolution of individual cells even if they are closely associated because the fluorescent nuclei are separated by a dark, non-fluorescent cytosol (Fig. 2).

**Figure 3:**
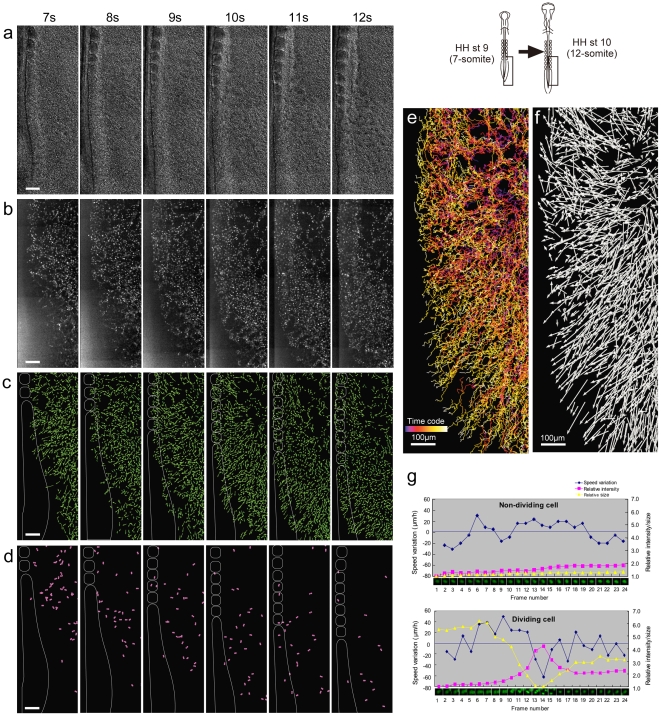
Dynamic analysis of EC movements within Tg(*tie1*:H2B-eYFP) embryos. (a) Cell tracking analysis was performed using Imaris software (Bitplane Inc.). A representative time lapse video (Video S4) was processed as follows: cropped time frames (5 through 67) corresponding to HH stages 9 to 11 and restricted region of interest to trunk level (posterior to somite 7, frame size is 500 mm ×1300 mm), and reduced noise using a median filter (3×3×3). Identified spots (i.e. ECs) that were over 5 µm size and thresholded for relative fluorescence levels resulted in ∼7500 of YFP^+^ spots to be selected and tracked using Brownian motion algorithm (minimum distance between each spot across time frame is 20 um). Every EC track was then manually corrected, resulting in ∼1,225 validated EC tracks. In the anterior vascular plexus area within the ROI, moving ECs appear to follow similar routes in a lateral to medial direction. Scale bar, 100 µm. (b) Pattern of displacement of the ECs within the vascular plexus. In the anterior level, ECs move horizontally from lateral to medial. The ECs around intermediate level move toward 45° posterior-medial direction. Most posterior level ECs tend to move perpendicular toward the tail. Scale bar, 100 µm. (c) Lineage track distribution of extra-embryonic- and embryonic-derived ECs. The tracked H2B-YFP^+^ ECs were classified into extra- and intra-embryonic origin groups beginning at HH9 (7ss) and traced to their destinations at HH stage 11 (12ss). The EC tracks displayed and analyzed were followed for at least 200 µm. Distribution pattern of the embryonic and extra-embryonic-derived ECs at HH stage 11 is correlated with original positional relationship at HH stage 9. The medial region of dorsal aorta is mainly derived from embryonic ECs whereas the lateral and posterior regions of the dorsal aorta are derived from extra-embryonic ECs. Scale bar, 100 µm. (d) New onset and division of ECs during development. The first spots of the entire EC tracks were classified manually into newly emerged and dividing ECs and then segregated into subgroups based that correlated their appearance with somite stage (ss) (i.e. ss 7: t2–12, ss 8: t13–23, ss 9: t24–33, ss 10: t34–43, ss 11: t44–53, ss 12: t54–63). The newly emerged cells and dividing ECs are highlighted with green and fused magenta spots, respectively. The majority of newly emerged and dividing ECs are found in the anterior lateral side in early stages and subsequently main places of the onset/divide shift from anterior lateral side to posterior lateral side and also axial areas along development. Scale bar, 100 µm. (e) Temporal trajectories are represented by color codes. Scale bar, 100 µm. (f) Overall displacement pattern of the ECs from 7-somite stage. Vectors visualize that the overall EC movement is lateral-to-medially biased. Scale bar, 100 µm. (g) Quantitative characterization of dividing ECs by tracking single eYFP signal. The eYFP signals taken every 5 minutes show decrease of their size and increase of fluorescent intensity when they undergo mitosis (upper panel). Migratory speed drops down significantly at the moment of mitosis. Non-dividing cell shows stable intensity, size and smaller speed variation (lower panel). Signal size and intensity are relative values. Speed variation represents speed (µm/hr) between each time. Plus and minus values indicate faster and slower speeds than average, respectively.

Time-lapse recordings show the ECs as they rearrange to form the vascular plexus and as they stream from the splanchnic mesoderm to form the two primitive dorsal aortae. To quantify the EC movement patterns and their cellular characteristics, we used Imaris software to identify and analyze individual ECs for several time-lapse videos. Fig. 3, representative of the quantified image data we acquired, highlights an area on the left side of the embryo that extends mediolaterally from the medial edge of the aorta to the extra-embryonic boundary and rostrocaudally from somite 7 to the vitelline artery. This time-lapse captured an image every 10 min from HH8 to HH13 (138 frames total) and then filtered for fluorescence intensity threshold levels and size of H2B-eYFP^+^ nuclei. The H2B-eYFP^+^ targets in every time frame were also validated and corrected manually. From this data set, ∼1,225 ECs were tracked to create cell migration tracks corresponding to individual cell movements. This sort of dynamic fate mapping provides complex data sets with information that can be quantitatively mined for EC positions and divisions, speed and route of movement, changes in nuclear shape and spacing that provides an overall, quantitative perspective of their interactions (Fig. 3).

By carefully adjusting the resolution and sensitivity of the optical system, it is possible to distinguish, via relative fluorescence intensity levels, whether a given H2B-eYFP EC is diploid or tetraploid. The intensity is fairly uniform during the G1 phase of the cell cycle, increases in S phase, and is twice as bright in G2 and early M phases, reflecting their increase in DNA content (Fig. 2h–j, 3e and Videos S2–S3). The brightest cells are typically those just entering mitosis; the daughter cells subsequently halve their relative fluorescence from that seen in G2 as the chromosomes are segregated. This predictable difference in relative fluorescence greatly facilitates computational analysis of EC proliferation.

The ECs undergo numerous cell divisions throughout vasculogenesis (Fig. 3d). The metaphase plate is oriented primarily perpendicular to the long axis of small, forming blood vessels, both inside and outside the embryo proper (Fig. 2, 3d and Videos S2, S3). In larger forming blood vessels such as the aorta and vitelline artery, the metaphase spindle shows no relationship to the vessels long axis. There is an obvious change in EC nuclear shape from round to prolate spheroid that accompanies maturation of both small and large vessels (Videos S3, S6). There is no apparent pattern or synchronicity of EC cell division during vasculogenesis (Fig. 3).

### ECs in vascular plexus move both independently and with underlying tissue

**Figure 4:**
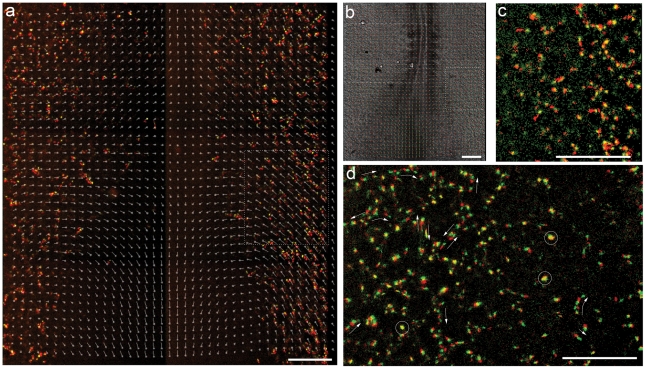
EC dynamics at the onset of vasculogenesis. The overall narrowing of the embryo appears as a tissue drift towards the midline, discernible by particle imaging velocimetry (PIV) analysis of the DIC image sequence (a) Motion of H2B-eYFP^+^ (HH stage 7, 5ss embryo) nuclei are shown in a somite-attached reference system by projecting four consecutive frames (the first three in red and the last one in yellow). The prevalent medial EC movement mostly reflects tissue drift, as the superimposed, PIV-derived displacement vectors indicate. (b) DIC image of the same specimen, with tissue motion vectors superimposed. (c) After removing tissue motion from the image sequence, displacements of H2B-eYFP^+^ nuclei appear random, without a prevalent directionality. Area shown in panel (c) is outlined with dotted lines in panels (a) and (b). (d) Cell-autonomous movement of nuclei an hour later in the nascent lateral network, outlined with a dashed line in panel (c). Two consecutive frames, separated by 8 minutes, are shown — the first as red, the second as green. Motile activity is inhomogeneous within the population: some nuclei do not move (appear as yellow, some are marked with circles), while most cells move in a chain-migration fashion (indicated by arrows). At this stage of vasculogenesis movement directions scatter widely: cell groups can move in opposite directions even along the same vascular segment. Scale bar: 200 µm.

During the earliest stages of vasculogenesis at the 5 somite stage (5ss; HH7) H2B-YFP^+^ nuclei appear randomly scattered in the lateral plate mesoderm, and then move medially (Fig. 3). Their medial movement is similar to the tissue movements of gastrulation. The overall narrowing of the embryo appears as a tissue drift towards the midline, discernible by particle imaging velocimetry (PIV) analysis of the DIC image sequence (Fig. 4). Subtracting the tissue-drift motion component from the total cellular displacements yields the “residual” or true autonomous cellular motility (Fig. 4; see [37],[43]), which appears random at the earliest stages. Thus, although primordial ECs are actively motile, the majority of their gross medial displacement at these stages is a consequence of tissue flow.

By stage HH9 (8ss) Tg(*tie1*:H2B-eYFP)^+^ ECs assemble into a reticular network lateral to the somites (Videos S2–S4). In the ectoderm, the major tissue movement at this developmental stage is an in-plane lateral to medial displacement of cells; tissue movement has ceased in the underlying mesoderm (Fig. 4a). There is an overall lateral-to-medial EC displacement of the entire vascular bed, but subtraction of this tissue-level motion continues to show that individual ECs also exhibit vigorous autonomous motility at widely varying rates and directions. ECs move by chain migration, and, when they divide, they form a metaphase plate perpendicular to the axis of migration, possibly reflecting that both migration and division are organized along extracellular matrix fibers (Videos S2–S4, S6). The chains of 3–5 ECs do not show strict directionality as they move along existing vascular polygons. Even within the same vascular segment, nearby EC chains can be observed moving in opposite directions, and individual cells can be seen switching movement directionality (Fig. 4; Videos S4, S5). The vascular polygons are continuously rearranged as the ECs increase in number from cell division, recruitment from the mesoderm, and immigration from the area opaca. This dynamism of the lateral vascular network seems surprising, given that at these stages the vascular polygons are composed of tubes, which conduct a rudimentary circulation.

### Dorsal aortae forms by individual and collective EC movements

During the developmental stages studied (HH6 to HH12) the dorsal aortae, sinus venosus and the vitelline arteries form by individual EC self-assembly and by coalescence or fusion of smaller-caliber vascular segments. The time-lapse epifluorescence microscopy recordings reveal that the medial and dorsal regions of the trunk dorsal aorta are derived mainly from embryonic ECs, whereas the lateral and posterior regions of the dorsal aorta are derived mainly from extra-embryonic ECs (Fig. 3c). The ECs within the aortae continue to proliferate, but do not show a preferred orientation of cell division. EC proliferation and ingression both account for the observed increase in length and girth of the aortae. The ECs adjacent to somites 1–5 either enter the aortae or migrate laterally to join the vascular plexus by stage 11, creating an avascular region lateral to the aortae. The avascular areas are transient and soon inundated by more ECs ingressing into the aorta from the vascular plexus.

The ability to trace eYFP-expressing nuclei and subtract the motion of the adjacent tissue allows the degree of “real” or autonomous cellular movement to be determined. This reveals that ECs move anterior (i.e. towards the heart, against the flow) in the dorsal aortae and ECs in the vitelline vessels move medially to join the aortae. The ECs in the forming trunk dorsal aortae appear to move in mirror-image helices: cells in the right aorta appear to move rostrally and clockwise; cells in the left aorta appear to move rostrally and counter-clockwise (Videos S7, S8). The medial helical motion results from the ingressing ECs pushing into the ventral regions of the aortae. The dorsal region of the dorsal aortae originates largely from somite derived ECs [38], but is difficult to unambiguously resolve in the z-axis over time with epifluorescence microscopy since we are imaging from the embryo's ventral surface with the modified New culture system.

**Figure 5:**
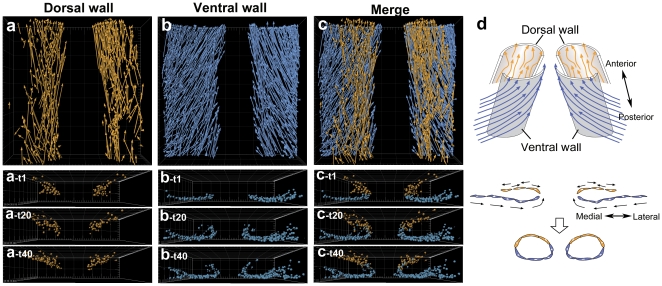
Different behaviors of dorsal and ventral walls of the forming dorsal aortae. (a) The 2P image data (see Videos S9 and S10) was subjected to cell tracking analysis. A total 1,218 EC tracks established were subdivided into dorsal and ventral halves of the dorsal aorta walls based on the first H2B-eYFP signal emergence position. The number of cells tracked in the dorsal and in the ventral walls was 444 and 874, respectively (a–c, and also see Videos S9 and S10). The dorsal wall ECs arise inside of the dorsal aortae and move diverse directions (a). The ventral wall ECs arise from the lateral side of the dorsal aortae and move medially in a unidirectional manner (b). 3D-reconstruction also reveals that these cells have different origins and behaviors (lower panels). The ventral dorsal aorta wall ECs, which are migrating from lateral mesoderm, curve toward dorsal and subsequently intercalate with the dorsal wall ECs at the boundary. In contrast to the ventral wall cells, the dorsal wall ECs do not move across the dorso-ventral boundary. These EC behaviors are illustrated in (d); upper panel is a ventral angle view, lower panel is transverse views).

An established method for imaging deep into living tissue, while inducing minimal fluorophore bleaching and cellular toxicity, involves two-photon laser scanning (2P) microscopy [39]. We used dynamic 2P microscopy to resolve the forming aortae along *xyz* axes to determine the cellular interactions responsible for the observed helical aorta movements in the developing Tg quail. Fig. 5 displays representative images from a typical 2P time-lapse (Videos S9, S10) of ECs forming the dorsal aortae adjacent to somites 2–6. 4D cell tracking of the ECs in the aortae resulted in a total of 1,218 manually validated EC tracks. We subdivided these tracks into dorsal and ventral halves of the dorsal aorta based on the position of the first appearance of the H2B-eYFP. Both dorsal (Fig. 5a,c) and ventral (Fig. 5b,c) half aortic ECs show similar anterior movement (Video S9). The ECs in the dorsal regions of the forming aortae exhibit variable mediolateral motions as they move rostrally; those in more ventral regions show significant lateral-to-medial movement as the course rostrally.

By viewing the 2P image sets in cross section, it is apparent that the ECs in the ventral half of the aortae move medially, seemingly pushed by the incoming splanchnic-derived ECs arriving from more lateral origins (Fig. 5a and Videos S9, S10). The ventral ECs then redirect dorsally as they approach the midline, soon intermixing with dorsal aortic ECs. The dorsal ECs exhibit varied mediolateral motions (Fig. 5a,c; top panel), but not the same systematic mediolateral cellular flow of the ventral aortic ECs. This mismatch in ventral and dorsal collective EC movement results in a “tractoring” motion that correlates and perhaps drives the motions of the paired aortae towards one another (Fig. 5b and Videos S9–S10). These distinct cell behaviors are illustrated in Fig. 5d. The tractoring motion may be driven by a mixture of large-scale tissue movements, by intercalary motions of the ECs, by changes in EC-ECM interactions, by resident aortic ECs undergoing cell division and cell orientation along the A–P axis, and/or by individual ECs continuing to enter into the aorta from the splanchnic and somatic mesoderm.

## Discussion

Transgenic Tg(*tie1*:H2B-eYFP) Coturnix quail, a warm-blooded amniote with a four-chambered heart, offer an accessible experimental system to record the dynamic events of vascular development. The Tg(*tie1*:H2B-eYFP) quail embryos fluorescently mark ECs in a specific and heritable manner, which permits dynamic analysis of vascular development with subcellular resolution. Live imaging of Tg quail embryos permits the dynamics of developmental processes such as individual and collective cell movements to be observed and quantified. New hypotheses of morphogenetic events often emerge from such unbiased observations, hypotheses that soon can be tested with molecular, cellular, or mechanical tools to understand embryogenesis in a quantitative manner.

Our dynamic imaging and computational analyses permit cellular characteristics such as proliferation, axis of division, and collective movement to be observed and characterized. The data show that EC cleavage during vasculogenesis is normally oriented perpendicular to the long axis of small vessels, as it is in mouse angiogenesis [40]. Perpendicular cell division favors vessel lengthening, as expected for the development of the vascular plexus in a growing embryo. Cell divisions during the formation of large vessels, such as the aorta or vitelline artery are not oriented along any axis. Large vessels grow in both length and breadth, likely requiring a continuum of cell division orientations. We did not observe synchronized EC proliferation in the developing vascular plexus, contrary to previous observations [41]. The imaging data reveal that individual ECs are highly motile, often moving as chains of cells, but the motility varies with position and stage as the ECs interact and coalesce to form vessels. The high spatial resolution of the nuclear fluorescence shows that large vessels such as the aortae and vitelline vessels move in predictable fashions as they take shape, and that the underlying EC movements are coordinated, but with considerable variability.

The use of the Tg(*tie1*:H2B-eYFP) to follow individual cells and brightfield imaging to follow overall tissue movements, provides a direct means to determine the relative roles that cell migration and tissue deformation play in the measured cell trajectories. The time-lapse videos reveal that primary vasculogenesis in the embryo involves vast tissue displacements and deformations (i.e. regression of the anterior intestinal portal, neural tube folding/closure, notochord extension, primitive streak regression). The presence of such large-scale motions raises the question of how the cellular and tissue level motions are related to one-another. It is possible that the significant cellular motility plays a role in the overall tissue motions, as has been observed in the convergent-extension movements. Where such movements have been studied in greatest detail (i.e. during frog gastrulation), mediolateral intercalary motions of a few cell diameters can more then double the length of a tissue [42]. Alternatively, it may be that the cell migrations are driven by nearby or distant tissue movements, which creates mechanical rearrangement of the cells or the extracellular matrix, and thereby drive the cellular events. Finally, it remains possible that the two are not causally related, but that the tissue morphogenetic movements merely create a moving platform in which the cellular motions take place. The use of the transgenic cellular labels reported here, together with tissue and ECM imaging techniques employed in the past [8], [19], [43], [44] offers a powerful means to decipher the causal relationships, if any, between cell and tissue motions.

The results presented here offer a dramatically improved and expanded view into the cell and tissue level events that build the paired dorsal aortae. The trunk dorsal aortae are formed by ECs coalescing to assemble large tubes. The ECs execute significant individual cellular motility, with all of the cells moving in a rostral direction at about HH11. The ECs in the dorsal regions of the forming aortae show variable mediolateral motions as they move rostrally; those in more ventral regions show significant lateral-to-medial movement as they course rostrally. These combined motions give the dorsal aortae the appearance of moving in mirror-image helical trajectories. The driving force(s) for the tissue level motions have yet to be defined [45]. One possibility is that the ECs derived from the splanchnic mesoderm stream into the ventral aspect of the developing aortae, while those derived from the somitic mesoderm stream into the dorsal aspect of the developing aortae. The significant cell movement into the ventral regions from the more lateral mesoderm may be a driving force to the biased motions.

The present results offer a powerful approach to the major challenge of studying the relative role(s) of the mechanical, molecular, and cellular mechanisms of vascular development. In past studies, the advantages of the molecular genetic tools available in mouse were counterbalanced by the limited experimental accessibility needed for imaging and perturbation studies. Avian embryos provide the needed accessibility, but few genetic resources. The creation of transgenic quail with labeled endothelia builds upon the important roles that quail and chicken embryos have played in previous studies of vascular development, using the developing embryo for tissue transplant and imaging experiments [4], [46]. Transgenic quail lines bring molecular genetic approaches to an accessible system with embryonic development and cardiogenesis that closely parallel human development. This offers the exciting prospect of a single experimental system that can be applied to problems that currently require parallel and non-equivalent experiments performed in a variety of lower and higher vertebrates.

## Materials and Methods

### Plasmid Construction

The original vector, pRRLsin.cPPT.Tie1p.eGFP.wpre, was a kind gift from Dr. Luigi Naldini [47]. Expression of the mouse *Tie1* promoter was previously characterized [48]. The human H2B histone tag, inserted into the pEGFP-N1 cloning vector (Clontech), was a generous gift from Dr. Geoffrey Wahl [36]. The eGFP cassette was removed from the pRRLsin.cPPT.Tie1p.eGFP.wpre vector with *Age*I and *BsrG*I and replaced with an 1116 bp *Nhe*I-*Not*I H2B-eYFP fragment using a blunt end ligation. The final plasmid vector, pLenti.*tie1*:H2B-eYFP (7,813 bp), was electroporated into DH10B competent *E. coli* (Invitrogen).

### Lentivirus Production

293FT cells (Invitrogen) were grown on gelatin coated plates and transfected with pLenti.*tie1*:H2B-eYFP using Lipofectamine 2000 along with the ViraPower Lentiviral Packaging Mix (Invitrogen) according to the manufacturer's protocol. Supernatants were collected at 24, 48 and 72 hours post-transfection, filtered at 0.45 µm and stored at −80°C until concentration. Supernatants were concentrated using Centricon Plus filter devices with a 30 kD MW cutoff (Millipore) according to the manufacturer's protocol. The resulting supernatants were ultracentrifuged at 50,000×g for 2 hr at 4°C. The lentiviral pellets were resuspended in DMEM (Mediatech) and stored at −80°C. The lentiviral titer was determined by infecting YSE2 (murine yolk sac endothelial) cells with serial dilutions of the concentrated virus. Only transfections resulting in lentiviral titers of at least 1×10^8^ transforming units/ml were used for blastoderm injection.

### Animal Care

All experimental methods and animal husbandry procedures were performed in accordance with the guidelines of the National Institutes of Health and with the approval of the Institutional Animal Care and Use Committee at the California Institute of Technology. Additional information on quail care and husbandry is available [49].

### Production of Transgenic Quail

Freshly laid quail eggs were collected daily and stored at 13°C for no longer than 2 weeks. On the day of injection, the eggs were laid on their sides at RT with the long axis of the egg parallel to the lab bench for a minimum of 2 hours. This position allowed the embryo to float to the highest point inside the shell. A small hole was cut in the eggshell directly over the embryo. 1–2 µl of concentrated lentivirus solution was injected into the subgerminal cavity of Stage X Japanese quail embryos. The eggs were filled with HBSS (pH 7.4) and sealed with a Steri-strip (3 M Health Care) and molten paraffin wax. The injected eggs were incubated at 37.5°C with 56% humidity for 16 days until hatching. A total of 184 embryos were injected with lentivirus. Of these, 4 embryos were successfully hatched (2.2%). After reaching sexual maturity, each of the four G0 mosaic founders was bred to a separate WT mate. Of the four founders, one produced transgenic offspring. This founder pair produced 129 eggs, five of which were positive for the transgene (3.8%). Three of these hatchlings were grown to adulthood and bred for experimental analysis.

For naming the transgenic quail, we followed the nomenclature guidelines established for other model organisms (http://zfin.org/cgi-bin/webdriver?MIval=aa-ZDB_home.apg).

### Analysis of Transgenic Quail

Screening G1 hatchlings for the presence of the transgene was conducted in two ways. First, PCR analysis was performed on genomic DNA isolated from the chorioallantoic membrane (CAM) of the eggshell after hatching. The CAM was scraped from the inside of the shell and digested overnight at 55°C in the presence of SDS and proteinase K. The genomic DNA was isolated using standard phenol/chloroform extraction protocols. 100 ng of genomic DNA was used to perform multiplex PCR with oligonucleotide primers designed against the H2B-YFP portion of the transgene along with chicken GAPDH as a housekeeping control. Second, the endothelial specific expression pattern of the *mTie1* promoter allowed us to screen the empty eggshells by illuminating them under an epifluorescence stereo dissecting microscope. The blood vessels of the CAM were clearly fluorescent in all three lines of transgenic quail. In addition, the highly vascular tissue at the thick follicle end of breast feathers from adolescent birds was also highly fluorescent in transgenic birds. Once transgenic birds had been identified by these methods, the number of transgene integrations was determined using Southern blot analysis. 5 ug of genomic DNA was digested with *Pst*I, separated on a 0.8% agarose gel and transferred to a nylon membrane. The blot was hybridized with a 646 bp ^32^P-labeled DNA probe against the woodchuck hepatitis virus posttranscriptional response element of the transgene.

### Cloning of *tie1*

Chicken total RNA was isolated from HH stage 13 embryos according to the manufacturer's instructions (RNeasy, Qiagen). 343 bp and 852 bp partial cDNA fragments of chicken *tie1* were obtained by RT-PCR using the following primers, which were designed based on the BBRC chicken EST database (http://www.chick.manchester.ac.uk/). 354 bp fragment, 5′-gctctagagcagccatcaagatgctgaag-3′ and 5′-caagatctgcagtggaataagcctgaga-3′, 852 bp fragment 5′-gctctagagtggtgtctacagtgccactt-3′and 5-gaagatcttatcaccagagaagcagtcca-3′. These fragments were then subcloned into the *Xba* I and *Bgl* II sites of pBluescript II-SK (Stratagene).

### Whole-mount in situ hybridization

Digoxigenin-labeled *TIE1* RNA probes (343 bp and 852 bp) were prepared according to the manufacturer's instructions (Roche). Quail embryos were fixed in 4% paraformaldehyde (PFA, Sigma) overnight at 4C. After washing in PBST (0.1% Tween-20 in PBS), the embryos were treated with 20 mg/ml proteinase K (Roche) in PBST for 10 min, re-fixed in 0.1% glutaraldehyde (Sigma)/4% PFA for 20 min, washed again in PBST three times, and pre-incubated with hybridization buffer (ULTRAhyb, Ambion) for 1 hour at 65C. The hybridization was carried out overnight at 65C in the hybridization buffer, containing a cocktail of the digoxigenin-labeled RNA probes. The embryos were washed three times in wash buffer 1 (50% formamide, 5xSSC, 1% SDS) for 30 min each at 65°C, and subsequently washed three times in wash buffer 2 (50% formamide, 2xSSC) for 30 min each at 65°C. After washing three times in MABT (0.1 M maleic acid (pH 7.4), 0.15 M NaCl, 0.1% tween20) for 5 min at room temperature, the embryos were pre-blocked in a two-step manner with 2% blocking reagent (Roche)/MABT and then with 2% blocking reagent, 20% fetal bovine serum (FBS)/MABT at room temperature for 1 hour each, followed by overnight incubation at 4°C with alkaline phosphatase-conjugated anti-digoxigenin-Fab fragments (1∶2000 dilution)(Roche). After washing in MABT eight times every 30 min, the embryos proceeded to three ten-minute washes in NTMT (100 mM Tris-HCl (pH 9.5), 100 mM NaCl, 50 mM MgCl2, 0.1% Tween20). In order to visualize the probe, the embryos were incubated overnight in 5 µl/ml NBT (Roche), 37.5 µl/ml BCIP (Roche) in NTMT at room temperature. The color reaction was terminated by washing three times in PBST and fixed in 0.1% glutaraldehyde/4% PFA for 30 min at 4°C.

### Whole-mount immunostaining

Embryos were fixed in 4% PFA/PBS overnight at 4°C and washed in PBST (0.1% triton-X100 in PBS) three times. After preblocking with 5% FBS, 0.2% bovine serum albumen (BSA)/PBST for 1 hour at room temperature, the embryos were incubated overnight at 4°C in a cocktail of QH1 mouse antibody (1∶1000 dilution) (Developmental Studies Hybridoma Bank) and anti EGFP rabbit antibody (1∶1000 dilution) (Clontech) in 5% FBS, 0.2% BSA/PBST, followed by washing six times for 30 min each in PBST. Subsequently, the embryos were blocked again with 5% FBS, 0.2% BSA/PBST for 30 min at room temperature, and incubated overnight at 4°C with a cocktail of anti mouse IgG-Alexa 688 goat antibody (1∶1000 dilution) (Clontech) and anti rabbit IgG-FITC donkey antibody (1∶500 dilution) (Abcam), and Hoechst (1∶10,000) (Invitrogen) followed by washing six times for 30 min each in PBST.

### In vitro imaging

Quail embryos, stage HH5 to HH12 (presomite to somite 17) were used in this study for dynamic analysis. We used whole-mount ex ovo avian embryo culture initially described by New [50] and modified as described in [44] to secure the quail embryo to its vitelline membrane. Wild-type and Tg(*tie1*:H2B-eYFP) quail embryos were incubated at 37°C in a humidified chamber to the desired Hamburger–Hamilton (HH) stages. Embryos were then dissected from the egg and mounted on filter paper rings ventral side up on a semi-solid mixture of agar/albumen. A custom-built chamber mounted on the microscope stage maintained the temperature at 37°C during imaging.

### Digital Time-Lapse Microscopy

The details of the digital time-lapse microscopy system have been fully described elsewhere [19], [43]. Briefly, using custom-written software, a computer-controlled wide field (10× objective) epifluorescent microscope (Leica DMR) workstation, equipped with motorized stage and cooled digital camera (Qlmaging Retiga 1300), is used to acquire 12-bit grayscale intensity images at multiple *xy* locations (fields) and focal planes (*z*-stacks). Automated switching of illumination modes and optical filters enables sequential acquisition of separate fluorescent wavelengths, in addition to brightfield or differential interference contrast (DIC) images. So, for one embryo, a single acquisition cycle or frame results in **nf nz ni** images, where nf is the number of fields (usually 6–10), nz is the number of focal planes (7–13), and **ni** is the number of illumination modes. The exposure time, typically on the order of 100–500 ms, depends on both the robustness (i.e. brightness and stability) of the particular fluorochrome(s) used and the camera sensitivity. In general, frame rates range from 5–15 per hour in this setup. For a typical 10-h experiment, upwards of 20,000 images (approx. 20 GB) are acquired. During an experiment, images are uploaded to a storage server (Xserv RAID, Apple, Inc.) for archiving and subsequent processing. To reduce a 3-D tiled image data set to a single focused plane, custom-written processing software automatically merges adjacent fields (i.e. mosaic-ing), and then collapses the merged focal planes using either a maximum local contrast algorithm or maximum through-plane grayscale intensity (i.e. z-projection), as is often done for confocal image sets. Typically, the local contrast method is used for brightfield or DIC images, and z-projection is used for fluorescent images. The resulting collapsed frames are then registered, using the center of the embryo as a reference location, thus correcting for any drift in the horizontal position of the embryo that may occur during an experiment, due to the nature of the ring culture method.

For the confocal and two-photon laser imaging, we used a Zeiss 510 META NLO microscope equipped with an EC plan-Neofluar 20×/0.5 objective at 900 nm (17.9%) excitation, to collect image sets that were 450×450×111 mm (3 mm interval) in xyz (n = 8). The xyz imaging cycle was typically taken 7.5 min for a total duration of 6.5 hours.

### Computational Analysis

Cell tracking analysis was performed using Imaris software (Bitplane Inc.) on time-lapse videos. Time frames from 5 through 67 based on somite numbers (stage 9 to 11) were cropped to a frame size of 500 µm ×1300 µm and the region of interest was restricted to the trunk level (posterior to somite 7). Noise reduction was accomplished using a 3×3×3 median filter. Labeled nuclei (spots) were tracked using a Brownian motion algorithm (the minimum distance between each spot across the time frame was 20 µm). In order to be tracked, each labeled nucleus (average diameter of 8–12 µm) was at least 5 µm in diameter with a minimum threshold level of 7500. Each automatically generated track was also reviewed manually. Cell displacement patterns were drawn using the displacement tracking mode of Imaris.

Levels on images were adjusted in Adobe Photoshop to match the collected intensity histogram to the full 8-bit output range.

### Tissue deformations

Tissue motion was extracted using the two-step particle image velocimetry (PIV) algorithm [35]. Briefly, images were divided into overlapping tiles. For each of these tiles their displacement is determined by cross-correlation analysis. The resulting displacement vectors were then interpolated and denoised by a thin-plate spline fit (Matlab). The resulting coarse displacement map was used to construct a second, higher resolution one. To reduce ambiguities associated with smaller tiles, cross correlations were evaluated only around positions predicted by the coarse map.

To remove tissue motion from the image sequence, we applied the following transformation. If h(x,t) is the brightness of pixel x at frame t, and u(x,t) denotes the PIV-derived (backward) displacement field between frames t and t-1, then the transformed image h' is given as h'(x,t) = h(x-u(x,t),t). By applying a series of such transformations, the original h(x,t), h(x, t+1), h(x, t+2), … image sequence was replaced by the h(x,t), h(xu(x,t+1),t+1), h(x-u(x,t+2)-u(x,t+1), t+2), … sequence.

## Supporting Information

Video S1 Low magnification dynamic imaging of Tg(*tie1*:H2B-eYFP^ct2^) quail embryo. Dynamic imaging of Tg(*tie1*:H2B-eYFP^ct2^) quail embryo using Leica DMR upright microscope in DIC and epifluorescence modes with a 5× objective for ∼36 hours every 13 minutes. Scale bar  = 600 mm.
(112.68 MB MOV)

Video S2 Time-lapse movies showing Tg(*tie1*:H2B-eYFP) cell nuclei surrounded by QH1+ plasma membrane in EC cells. Tg(*tie1*:H2B-eYFP) quail embryos were injected with QH1-A647 at stage 7–8 and time-lapse captured every 13 minutes for 8.5 hours until 15 somites (stage 11). The images were acquired on the upright microscope with the dorsal side against the EC Agar culture using the 10× objective and 2×2 binning. 2×5×9 Mosaic. Composite movie of SV 2b–c showing Tg(*tie1*:H2B-eYFP) cell nuclei (green) surrounded by QH1+ plasma membrane in EC cells.
(13.11 MB MOV)

Video S3 Composite movie showing Tg(*tie1*:H2B-eYFP) cell nuclei (green) surrounded by QH1+ plasma membrane in EC cells.
(26.94 MB MOV)

Video S4 Imaris based cell tracking (*xyt*) of Tg(*tie1*:H2B-eYFP) time-lapse video. ECs arose at somite stage 7, 8, 9, 10, 11 are labeled by cyan, magenta, green, yellow, orange, respectively. The ECs intermingle each other during migration from lateral to medial, thereby give rise to vascular network (also see Video S5).
(45.77 MB MOV)

Video S5 Polygons are continuously rearranged. a) The area shown moves with the tissue, so the motion seen is autonomous. During a 10 h long time period, from HH9 (8 somites) until HH12 (15 somites), the avascular areas disappear and re-form at other locations.
(0.17 MB MOV)

Video S6 Time-lapse of Tg(*tie1*:H2B-eYFP) embryo shows anteriomedial rotation of newly assembled dorsal aortae. Embryos injected with QH1-A647 and imaged on the upright microscope with the dorsal side against the EC Agar culture using the 10× objective and 2×2 binning. The time lapse records strong H2B::eYFP signal starting at Stage 7–8. All of these embryos develop normally ∼15 hours. Q715: Index T000 to T033 ∼10.5 minute time points using 2×3×5 Mosaic; then Index T034 to T91 ∼10 minute time points using 2×3×7 Mosaic.
(48.06 MB MOV)

Video S7 Cells move upstream (towards the heart, against the flow) and circulate within the walls of the dorsal aortae. Moving projection of 4 frames, past positions are dimmer, actual position is bright. Colored z-projection, red: ventral, blue: dorsal. Distance between layers colored purple and blue is 40–80 mm (dz = 40 mm). Cells in the ventral cylinder surface (purple) move medially more than cells in the dorsal surface (blue). Movie of 19 frames (2.5 h), in a somite-attached frame. Movie with moving projection.
(0.23 MB MOV)

Video S8 Anteriomedial rotation of the dorsal aortae. Moving projection of 4 frames, each separated by 10 minutes. Dorsal and a ventral image planes, 50 um apart, are shown in the left and right panels, respectively. In the central panel the two planes are superimposed with color-coding (red: ventral, blue: dorsal). Cells move upstream (towards the heart, against the blood flow) and circulate within the walls of the dorsal aortae: Cells in the ventral cylinder surface (red) move medially more than cells in the dorsal surface (blue). Images are shown in a somite-attached frame of reference.
(0.44 MB MOV)

Video S9 2P microscopy of forming dorsal aortae from stage 11 through 12 (longitudinal view). The time-lapse was acquired by Zeiss 510 META NLO as following: Objective: EC plan-Neofluar 20×/0.5, Chameleon: 900 nm (17.9%), Z-stack size: 111 um (3 um interval), Imaging cycle: 7.5 min, Total duration: 6.5 hours. Bright signals in the midline are non-specific excitation of yolk by the 2P excitation. Movie corresponds with data in Figure 5. Bottom. 4D EC tracking of the dorsal and ventral walls of the dorsal aortae (longitudinal view). Dorsal and ventral wall endothelial cells are indicated by orange and cyan, respectively. Movie corresponds with data in Figure 5.
(17.53 MB MOV)

Video S10 Top. 2P microscopy of forming dorsal aortae from stage 11 through 12 (transverse view). The time-lapse was acquired by Zeiss 510 META NLO as following: Objective: EC plan-Neofluar 20×/0.5, Chameleon: 900 nm (17.9%), Z-stack size: 111 um (3 um interval), Imaging cycle: 7.5 min, Total duration: 6.5 hours. Bright signals in the midline are non-specific excitation of yolk by the 2P excitation. Movie corresponds with data in Figure 5. Bottom. 4D EC tracking of the dorsal and ventral walls of the dorsal aortae (transverse view). Movie corresponds with data in Figure 5.
(5.12 MB MOV)
